# A new effective method for estimating missing values in the sequence data prior to phylogenetic analysis

**Published:** 2007-02-01

**Authors:** Abdoulaye Baniré Diallo, François-Joseph Lapointe, Vladimir Makarenkov

**Affiliations:** 1 Département d’informatique, Université du Québec à Montréal, C.P. 8888, Succ. Centre-Ville, Montréal (Québec), H3C 3P8, Canada. banire(at)lacim.uqam.ca; makarenkov.vladimir(at)uqam.ca; 2 Département de sciences biologiques, Université de Montréal, C.P. 6128, Succ. Centre-ville, Montréal (Québec), H3C 3J7, Canada. francois-joseph.lapointe(at)umontreal.ca

## Abstract

In this article we address the problem of phylogenetic inference from nucleic acid data containing missing bases. We introduce a new effective approach, called “Probabilistic estimation of missing values” (PEMV), allowing one to estimate unknown nucleotides prior to computing the evolutionary distances between them. We show that the new method improves the accuracy of phylogenetic inference compared to the existing methods “Ignoring Missing Sites” (IMS), “Proportional Distribution of Missing and Ambiguous Bases” (PDMAB) included in the PAUP software [26]. The proposed strategy for estimating missing nucleotides is based on probabilistic formulae developed in the framework of the Jukes-Cantor [10] and Kimura 2-parameter [11] models. The relative performances of the new method were assessed through simulations carried out with the SeqGen program [20], for data generation, and the Bio NJ method [7], for inferring phylogenies. We also compared the new method to the DNAML program [5] and “Matrix Representation using Parsimony” (MRP) [13], [19] considering an example of 66 eutherian mammals originally analyzed in [17].

## Introduction

The presence of missing and ambiguous data in the sequences of nucleotides is one of the major problems in phylogenetic analysis of fossil taxa as well as of combined datasets (e.g. different genes, morphology) that do not include identical sets of taxa [23], [29]. Huelsenbeck [9] and Makarenkov and Lapointe [16] pointed out that the presence of taxa comprising a big percentage of unknown nucleotides might considerably deteriorate the accuracy of the phylogenetic analysis. Obviously, gaps, which are caused by deletions and insertions of nucleotides, should not be considered as missing data. The following questions are often raised: (1) Should we consider or ignore sequences comprising missing data in the phylogenetic analysis? (2) Is it necessary to consider sites with unknown entries? In this study, we are mostly interested in the second question. The popular PAUP software [26] includes two methods for computing evolutionary distances between species from incomplete sequence data. The first method, called IMS (“Ignoring missing sites”) is the most commonly used. It proceeds by the elimination of incomplete sites while computing evolutionary distances. According to Wiens [29], such an approach represents a viable solution only for long sequences. Philippe et al. [18] pointed out that in case of long sequences, the sites with missing data can be omitted because of the presence of a sufficient number of nucleotides [30]. The second method included in PAUP, called PDMAB (“Proportional distribution of missing and ambiguous bases”), computes evolutionary distances taking into account missing bases in the 2 sequences while computing the pairwise distance. PDMAB assigns values corresponding to the missing characters comparing sequences on the one-to-one basis. In our opinion, it would be more accurate to compute the probability of each of the missing DNA nucleotides to be A, C, G or T, taking into account the whole set of aligned sequences. Thus, the new method will consider all available information associated to the similarities among the sequences, the nucleotide frequencies and the characters present in a specific site. Hence, we propose a new method, called PEMV (“Probabilistic estimation of missing values”), which estimates the identities of all missing bases prior to computing pairwise distances between species. This estimation tries to correct the weakened signal caused by the presence of missing data [30]. To estimate a missing base, the new method proceeds by computing a similarity score between the sequence comprising the missing base and all other sequences. A probabilistic approach is used to determine the likelihood of an unknown base to be either A, C, G or T for DNA sequences, or A, C, G or U for RNA sequences. The main idea of the new method is to identify the probabilities of each missing data to be a particular nucleotide character and then to use them for computing the interspecies distances. Moreover, the obtained probabilities can be incorporated into the distance computation formulas used in the framework of different evolutionary models. We show how this method can be applied in the framework of the Jukes-Cantor [10] and Kimura 2-parameter [11] models. In the next two sections we introduce the new method for estimating missing entries in sequence data and compare it to the two methods available in the PAUP package. Then, we discuss the results provided by the three competing methods in a simulation study carried out with DNA sequences of different lengths, containing different percentages of missing bases (datasets were generated by the SeqGen program [20]). The accuracy of the phylogenetic inference is assessed by means of the Robinson and Foulds topological distance [21]. The BioNJ method by Gascuel [7], which was shown to provide better results for sequence data than the popular NJ method [22], was used in our simulations to reconstruct phylogenies. In the application section we explore how PEMV copes with partial gene data comprising 15 nuclear genes of 64 placental and 2 marsupial species comparing it to the Maximum Likelihood (DNAML) [5] and Matrix Tree Representation (MRP) [1], [13], [19] methods. Note that the problem of missing nucleotides is mostly relevant for the distance-based methods. Maximum Likelihood and Maximum Parsimony methods consider missing bases and take them implicitly into account in the computation process.

## Probabilistic estimation of missing values

The new method for estimating unknown bases in nucleotide sequences, PEMV, is described here in the framework of the Jukes-Cantor [10] and Kimura [11] models of sequence evolution. The Jukes-Cantor model assumes that all nucleotides A, C, G and T have the same frequency and that all substitutions are equally likely (e.g. the probability of transition is equal to that of transversion). To compute evolutionary distances between any pair of sequences within this model, the following correction formula is used: *d* = −3/4 *ln* (1−4/3**D**), where **D** is the observed distance, computed as the number of mismatches between pairs of sequences divided by the number of compared sites. In the Kimura 2-parameter model, the following formula is used: $d=-1/2\;ln\;((1-2P-Q)\sqrt{\left(1-2Q\right)})$ to compute the distance between a pair of sequences, where *P* is the transitional and *Q* is the transversional difference between them. This model gives better distance estimates than the Jukes-Cantor model when the transition and transversion rates are different.

Assume that the base *k* in the sequence *i* is missing. To compute the distance between the sequence *i* and all other considered sequences, PEMV estimates, using Equation 1 below, the probabilities *P_ik_*(A), *P_ik_*(C), *P_ik_*(G) and *P_ik_*(T) to have respectively the nucleotide A, C, G or T at site *k* of the sequence *i*. The probability that an unknown base at site *k* of the sequence *i* is a specific nucleotide depends on the number of sequences having this nucleotide at this site as well as on the distance (computed ignoring the missing sites) between *i* and all other considered sequences having known nucleotides at site *k*. First, we calculate the similarity score δ between all observed sequences while ignoring missing data. For a pair of aligned sequences, this score is equal to the number of matches between homologous nucleotides divided by the number of comparable sites.

(1) $$\begin{array}{l} P_{ik}(\text{A})=\frac{1}{N_{k}}\left(\sum_{\left\{j|C_{jk}=\text{A}\right\}}\delta_{ij}+\frac{1}{3}\sum_{\left\{\begin{array}{l} j|C_{jk}\neq \text{A}\\ \text{and }C_{jk}\neq ? \end{array}\right\}}\left(1-\delta_{ij}\right)\right)\\ P_{ik}(\text{C})=\frac{1}{N_{k}}\left(\sum_{\left\{j|C_{jk}=\text{C}\right\}}\delta_{ij}+\frac{1}{3}\sum_{\left\{\begin{array}{l} j|C_{jk}\neq \text{C}\\ \text{and }C_{jk}\neq ? \end{array}\right\}}\left(1-\delta_{ij}\right)\right)\\ P_{ik}(\text{C})=\frac{1}{N_{k}}\left(\sum_{\left\{j|C_{jk}=\text{G}\right\}}\delta_{ij}+\frac{1}{3}\sum_{\left\{\begin{array}{l} j|C_{jk}\neq \text{G}\\ \text{and }C_{jk}\neq ? \end{array}\right\}}\left(1-\delta_{ij}\right)\right)\\ P_{ik}(\text{C})=\frac{1}{N_{k}}\left(\sum_{\left\{j|C_{jk}=\text{T}\right\}}\delta_{ij}+\frac{1}{3}\sum_{\left\{\begin{array}{l} j|C_{jk}\neq \text{T}\\ \text{and }C_{jk}\neq ? \end{array}\right\}}\left(1-\delta_{ij}\right)\right), \end{array}$$

where *N_k_* – is the number of known bases at site *k* (i.e. column *k*) of the considered aligned sequences; δ*_ij_* – is the similarity score between the sequences *i* and *j* computed ignoring missing sites, **C** – is the matrix of aligned DNA sequences; *P_ik_*(A), *P_ik_*(C), *P_ik_*(G) and *P_ik_*(T) – are the probabilities of the missing base *k* of the sequence *i* to be A, C, G or T, respectively.

The following Theorem characterizing the probabilities *P_ik_*(A), *P_ik_*(C), *P_ik_*(G) and *P_ik_*(T), can be stated:

### Theorem 1

For any sequence i, and any site k of the matrix **C,** such that C_ik_ is a missing nucleotide, the following equality holds: P_ik_(A) + P_ik_(C) + P_ik_(G) + P_ik_(T) = 1.

The proof of this theorem is presented in Appendix.

Once the different probabilities *P_ik_* are obtained, we can compute the matrix of distances **D** between all given sequences applying Equation 2. The *PEMV* distance *d* within the Jukes-Cantor model is computed as follows:

(2) $$d_{ij}=\frac{N_{ij}^{c}-N_{ij}^{m}+\sum_{\left\{\begin{array}{l} k|C_{ik}\neq ?\\ \text{or }C_{jk}\neq ? \end{array}\right\}}\left(1-P_{ij}^{k}\right)}{N},$$

where *d_ij_* – is the distance between the sequences *i* and *j*, *N* – is the total number of sites (i.e. number of columns in the matrix **C** that have at least one known nucleotide); *N_ij_^m^*– is the number of matches between homologous nucleotides in the sequences *i* and *j; N_ij_^c^*– is the number of comparable pairs of nucleotides in the sequences *i* and *j* (i.e. when both nucleotides are known in the homologous sites of *i* and *j*); *P_ij_^k^* – is the probability to have a pair of identical nucleotides at site *k* of the sequences *i* and *j*.

When both nucleotides at site *k* of the sequences *i* and *j* are missing, *P_ij_^k^* is computed as follows:

(3) $$\begin{array}{l} P_{ij}^{k}=P_{ik}(\text{A})P_{jk}(\text{A})+P_{ik}(\text{C})P_{jk}(\text{C})+\\ P_{ik}(\text{G})P_{jk}(\text{G})+P_{ik}(\text{T})P_{jk}(\text{T}), \end{array}$$

where the values of *P_ik_* and *P_jk_* are determined according to Equation 1. Then, the Jukes-Cantor logarithmic transformation can be applied to *d_ij_* to transform it into the corrected distance.

In the case of the Kimura 2-parameter model, we have first to compute the probabilities *P_ij_^k^* for each missing nucleotide of the matrix **C**, and then, calculate, using Equation 4, the transitional difference *P*(*i,j*) and the transversional difference *Q*(*i,j*) prior to applying the Kimura logarithmic transformation:

(4) $$\begin{array}{l} P(i,j)=\frac{P^{\prime }(i,j)+\sum_{\left\{\begin{array}{l} k|C_{ik}\neq ?\\ \text{or }C_{jk}\neq ? \end{array}\right\}}P^{\prime }(i,j,k)}{N}\\ Q(i,j)=\frac{Q^{\prime }(i,j)+\sum_{\left\{\begin{array}{l} k|C_{ik}\neq ?\\ \text{or }C_{jk}\neq ? \end{array}\right\}}Q^{\prime }(i,j,k)}{N}, \end{array}$$

where *P*′ (*i,j*) – is the number of transitions between the sequences *i* and *j* computed ignoring missing sites; *P*′ (*i*,*j*,*k*) – is the probability of transition between the sequences *i* and *j* at site *k* when the nucleotide at site *k* is missing either in *i* or in *j* (e.g. if the nucleotide at site *k* of the sequence *i* is A and the corresponding nucleotide in *j* is missing, we have to evaluate the probability that the missing base of the sequence *j* is G); *Q*′ (*i*,*j*) – is the number of transversions between the sequences *i* and *j* computed ignoring missing sites; *Q*′ (*i*,*j*,*k*) – is the probability of transversion between the sequences *i* and *j* at site *k* when the nucleotide at site *k* is missing either in *i* or in *j* (e.g. if the nucleotide at site *k* of the sequence *i* is A and the corresponding nucleotide in *j* is missing, we have to evaluate the probability that the missing base of the sequence *j* is either C or T).

When both nucleotides at site *k* of the sequences *i* and *j* are missing, *P*′ (*i*,*j*,*k*) and *Q*′ (*i*,*j*,*k*) are computed as follows:

(5) $$\begin{array}{l} P^{\prime }(i,j,k)=P_{ik}(\text{A})P_{jk}(\text{G})+P_{ik}(\text{C})P_{jk}(\text{T})+\\ P_{ik}(\text{G})P_{jk}(\text{A})+P_{ik}(\text{T})P_{jk}(\text{C}),\\ Q^{\prime }(i,j,k)=P_{ik}(\text{A})(P_{jk}(\text{C})+P_{jk}(\text{T}))+\\ P_{ik}(\text{C})(P_{jk}(\text{A})+P_{jk}(\text{G}))+\\ P_{ik}(\text{G})(P_{jk}(\text{C})+P_{jk}(\text{T}))+\\ P_{ik}(\text{T})(P_{jk}(\text{A})+P_{jk}(\text{G})). \end{array}$$

## A numerical example

In this section, we present a numerical example to show the difference between the new sequence-to-distance transformation method (PEMV) and the two methods available in PAUP (IMS and PDMAB). We use the dataset reported in Table 1 comprising 3 sequences of 8 nucleotides each (the character ‘−’ represents a missing base).

We apply the three transformation methods to the matrix **C** (Table 1) to compute the distances *d*_12_, *d*_13_ and *d*_23_ using the Kimura 2-parameter model. We explain how the computation should be carried out in the case of IMS, PDMAB, and PEMV:

1. Using the IMS method, which ignores missing sites when computing *d*_12_, we consider only the 6 complete sites in the sequences 1 and 2. There is one difference between them due to a transversion. Under the Kimura 2-parameter model the distance *d*_12_ is equal to 0.1925. In the same way, we determine that *d*_13_ equals 0.4479 and *d*_23_ equals 0.3639.
2. Using the PDMAB method to compute *d*_12_, we first distribute missing bases according to the unambiguous changes between the two sequences (see the documentation of PAUP [26] for more details). When computing the distance between the sequences 1 and 2, we consider all pairs of corresponding bases such that the nucleotide in the sequence 1 is G (missing nucleotide must be compared to G). Two such pairs of bases, GG and GT, can be identified. According to the method, the probability to have the nucleotide either G or T in the site 5 of the sequence 2 will be 1 (0.5 for G and 0.5 for T). On the other hand, the value of 4 for AA is obtained by summing 3 (coming from the three pairs of AA appearing in the corresponding sites of the sequences 1 and 2) and 1 (coming from the comparison of a missing base in the site 8 of the sequence 1 and the nucleotide A in the same site of the sequence 2; the missing base of the sequence 1 should be equal to A according too PDMAB). Proceeding in the same manner, we compute the distribution matrix reported in Table 2.
  Analyzing Table 2, we notice that the transitional rate is 0 and the transversional rate is 1.5 (only for G to T). Therefore, the evolutionary distance between the sequences 1 and 2 equals to 0.2213 within the Kimura 2-parameter model. In the same way, we obtain that *d*_13_ equals 0.4052 and *d*_23_ is 0.3639.
3. Using the new method, PEMV, we have first to determine the probabilities that each missing base is either A, C, G or T. To do so, we proceed by computing the similarity score *δ*, considering only the complete sites. Thus, the values *δ*_12_ = 5/6, *δ*_13_ = 2/3 and *δ*_23_ = 5/7 are obtained. Then, we compute the number of bases present at sites containing missing data. Finally, we determine the probabilities *P*_25_ (A), *P*_25_(C), *P*_25_(G) and *P*_25_(T) using Equation 1. For instance, *P*_25_(A) is the probability that the missing nucleotide at site 5 of the sequence 2 is A. The computation is done as follows:

$$\begin{array}{l} P_{25}(\text{A})=1/3^{*}(1-5/6)=1/18,\\ P_{25}(\text{C})=1/3^{*}(1-5/6)=1/18,\\ P_{25}(\text{G})=5/6,P_{25}(\text{T})=1/3^{*}(1-5/6)=1/18. \end{array}$$

Similarly, we calculate the probabilities for the missing site 8 in the sequence 1:

$$\begin{array}{l} P_{18}(\text{A})={\left(1/2\right)}^{*}(5/6)+\\ {\left(1/2\right)}^{*}{\left(1/3\right)}^{*}(1-2/3)=17/36,\\ P_{18}(\text{C})={\left(1/2\right)}^{*}{\left(1/3\right)}^{*}(1-5/6)+\\ {\left(1/2\right)}^{*}(2/3)=13/36,\\ P_{18}(\text{G})=P_{18}(\text{T})={\left(1/2\right)}^{*}{\left(1/3\right)}^{*}(1-5/6)+\\ {\left(1/2\right)}^{*}{\left(1/3\right)}^{*}(1-2/3)=1/12. \end{array}$$

Once these probabilities are known, we compute the distance *d*_12_ using Equation 2. Here, we have 0 + 1/18 + 1/12 transitions and 1 + 1/18 + 1/18 + 13/36 + 1/12 transversions between the sequences 1 and 2. Thus, in the framework of the Kimura 2-parameter model, the distance between them is 0.2199. Similarly, we determine that *d*_13_ equals 0.436 (with 1 + 1/9 + 1/12 transitions and 1 + 1/9 + 1/9 + 17/36 + 1/12 transversions) and *d*_23_ equals 0.362 (Equation 5).

## Simulation design

A Monte Carlo study has been conducted to test the ability of the new method to recover correct phylogenies. We examined how PEMV performed depending on the length of the DNA sequences and the percentage of missing nucleotides. The simulations described in this article were conducted in the framework of the Kimura 2-parameter model. The results were obtained from simulations carried out with 1000 random binary phylogenetic trees with 8, 16, 24 and 32 leaves. In each case, a true tree topology, denoted *T*, was obtained using the random tree generation procedure proposed by Kuhner and Felsenstein [12].

The branch lengths of the true tree were computed using an exponential distribution. Following the approach of Guindon and Gascuel [8], we added some noise to the branches of the true phylogeny to create a deviation from the molecular clock hypothesis. All the branch lengths of *T* were multiplied by 1 + *ax*, where the variable *x* was obtained from a standard exponential distribution (*P*(*x* > *k*) = exp (−*k*)). The constant *a* is a tuning factor for the deviation intensity. Following the suggestion of Guindon and Gascuel [8], the value of *a* was fixed to 0.8. The random trees generated by this procedure are assumed to have the depth of *O*(log (*n*)), where *n* is the number of species (i.e. number of leaves in a binary phylogenetic tree). The source code of our tree generation program, written in C, is available at the following website: http://www.labunix.uqam.ca/~makarenv/tree_generation.cpp.

The random trees were then submitted to the SeqGen program [20] to simulate sequence evolution along their branches. We used SeqGen to obtain the aligned sequences of the length *l* (*l* = 125 and 500 bases) in the framework of the Kimura 2-parameter model [11]. To simulate missing data in the aligned sequences, we carried out two experiments following the strategies described by Wiens [30]. They differ by the way of distributing missing bases in the aligned sequences. The first strategy consists of removing at random a fixed percentage of nucleotides from the observed sequence, whereas the second strategy, which is certainly more realistic from a genomic point of view, consists of the random elimination of blocks of nucleotides of different sizes. In this paper, we processed data with 0 to 50% of missing bases. The obtained sequences were submitted to the three competing methods for computing evolutionary distances. For each distance matrix provided by IMS, PDMAB and PEMV, we inferred a phylogenetic tree *T*′ using the BioNJ algorithm [7].

The phylogeny *T*′ was then compared to the true phylogeny *T* using the Robinson and Foulds topological distance [21]. The Robinson and Foulds distance between two phylogenetic trees is the minimum number of operations, consisting of merging and splitting internal nodes, which are necessary to transform one tree into another. This distance was computed as percentage of its maximum value, which is 2*n*-6 for a phylogenetic tree with *n* leaves. The lower this value, the closer the obtained tree *T*′ to the true phylogeny *T*. Thus, in this simulation study we were able to evaluate the relative topology performance of the distance generation methods IMS, PDMAB and PEMV depending on the number of species, sequence length and percentage of missing nucleotides.

## Simulation results

In this section, we present the results of the simulations comparing the three methods for computing evolutionary distances. Sequence datasets for 8, 16, 24 and 32 taxa were generated. For each dataset, we tested the performance of the three methods depending on the sequence length (for sequences with 125 and 500 nucleotides) and the percentage of missing bases (ranging from 0 to 50%). Here, we present the results obtained with randomly distributed blocks of missing nucleotides because such a distribution of missing sites better reflects a biological reality. It is worth noting that the results obtained with the randomly removed nucleotides, that were not block-like distributed, were very similar.

**Figure 1:**
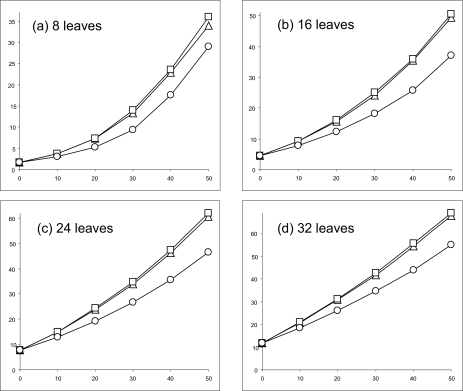
Mean topological recovery values obtained for 1000 random phylogenetic trees with 125 nucleotides. The percentage of missing bases varies from 0 to 50% (abscissa axis). The curves represent the variation of the Robinson and Foulds topological distance for the methods IMS (▵), PDMAB (□) and PEMV (○). The influence of the number of leaves is illustrated on the four panels: (a) 8 leaves, (b) 16 leaves, (c) 24 leaves, and (d) 32 leaves.

**Figure 2:**
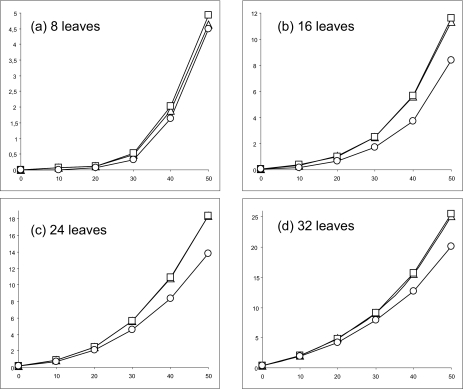
Mean topological recovery values obtained for 1000 random phylogenetic trees with 500 nucleotides. The percentage of missing bases varies from 0 to 50% (abscissa axis). The curves represent the variation of the Robinson and Foulds topological distance for the methods IMS (▵), PDMAB (□) and PEMV (○). The influence of the number of leaves is illustrated on the four panels: (a) 8 leaves, (b) 16 leaves, (c) 24 leaves, and (d) 32 leaves.

Figures 1 and 2 present the results produced by the three competing methods for the sequences with 125 and 500 nucleotides, respectively. First, for the phylogenies with 8, 16, 24 and 32 leaves PEMV clearly outperformed the PAUP methods (IMS and PDMAB) when the percentage of missing data was large. Second, the results obtained with IMS were very similar to those given by PDMAB, especially for the datasets with 16, 24, and 32 taxa. Third, only for 8 and 16 taxa and 500-nucleotide sequences (Figures 2a and 2b) did the three methods have similar performances (with a slight advantage for PEMV for 30 to 50% of missing data). In the latter case, it would be preferable to apply IMS, which is the simplest and the fastest of the three competing methods.

Obviously, the Robinson and Foulds topological distance increases when the number of taxa increases; this well-known trend shows up for all three methods. The Robinson and Foulds distance decreases when the length of sequences increases. The latter trend holds even for larger percentages of missing bases (a similar trend has been also reported by Wiens [30]). It is worth noting that the values of the Robinson and Foulds distance do not always equal zero with 0% of missing bases (especially for short sequences). This bias is due to the well-known problem of the estimation of short branches in phylogenies.

To assess whether the observed differences among the Robinson and Foulds distances corresponding to IMS, PDMAB and PEMV are statistically significant, we carried out the ANOVA test. The Null Hypothesis *H*_0_ for the ANOVA (F-test) was as follows: μ_IMS_ = μ_PDMAD_ = μ_PEMV_; where μ were the means of the corresponding Robinson and Foulds distances. The related P-values are indicated in Table 3. Thus, considering the level of significance α = 0.05, the differences depicted in Figures 1 and 2 were significant for the sequences with 125 bases (except the case of 8 taxa and 10% of missing data). For the sequences with 500 bases, the differences in the obtained results were significant for the cases of 16, 24 and 32 taxa when the percentage of missing nucleotides was above 10%. Since the performances of IMS and PDMAB were very similar, the ANOVA test basically consisted of measuring the difference between PEMV and the best of IMS and PDMAB.

Note that we also conducted the analysis of the distances obtained by the three competing methods before applying BioNJ. We compared these distances to the original distances (computed with the complete sequences) using the Pearson correlation coefficient. The obtained results did not show any well-established difference between the three methods. However, it is worth noting that PEMV generally outperformed IMS and PDMAB for the sequences with 125 bases but became less accurate for the sequences with 500 bases. The latter drawback can be due to an over-estimation of some of the distances by PEMV in the situation when there is a necessary amount of known homologous nucleotides to compute the distances.

## Inferring a phylogeny for a set of 66 mammalian species

In this section we apply PEMV to reconstruct a mammalian phylogeny from a segment of 15 nuclear genes of 64 placental and two marsupial species. The original phylogeny *T* for these species is presented in Figure 1 in [17]. The GenBank sequences with the accession numbers AY011125-AY012154 are considered in this study; the 15 selected genes are available for a various number of species (52 to 64).

Phylogenetic trees from multiple genes can be obtained using two fundamentally different approaches. In the first one, genetic sequences are concatenated into a single alignment (supermatrix) that is then submitted to a tree reconstruction method to generate a species tree [25]. As the concatenated genes do not always cover the same set of species, the blocks of missing nucleotides are present in the data [29]. In the second approach, the phylogenies are inferred separately from each gene and their supertree is computed to represent the phylogeny defined on the complete set of species [25].

Here we explore the ability of PEMV to infer trees from partial gene data. We compared PEMV to a supermatrix approach using DNAML [5] and to the well-known supertree method “Matrix Representation using Parsimony” (MRP). The DNAML program from the PHYLIP package implements the maximum likelihood method for DNA sequences [5]. MRP remains by far the most popular supertree method, owing to a combination of historical precedence coupled with universal applicability and good performance, producing well-resolved and usually accurate supertrees [2]. MRP was proposed by Loomis and Smith [13] and later refined independently by Baum [1] and Ragan [19]. Its implementation available in the Clann [3] program was used in this study. Note that as Clann only implements the MRP coding, we also used PAUP [26] to process the resulting matrix.

The data for 15 considered genes were aligned using CLUSTAL-X [28]. For the MRP analysis, the 15 gene trees were computed using the DNAML program. In all cases DNAML was used with the heuristic search, with the NNI branch swapping and the HKY model of nucleotide substitution, as suggested by Murphy et al. [17]. The best tree was always selected. The Kimura 2-parameter model of PEMV and NJ [22] were used.

We started the simulations reconstructing trees from three randomly chosen genes (out of 15), adding to them the other randomly chosen genes one-by-one until the concatenated dataset contained 15 genes (including 9702 sites in total). This procedure was repeated 100 times for PEMV and MRP, and 30 times for DNAML. The average Robinson and Foulds topological distance was computed between each tree obtained and the true phylogeny *T* (i.e. the tree given by Murphy et al. [17]).

**Figure 3:**
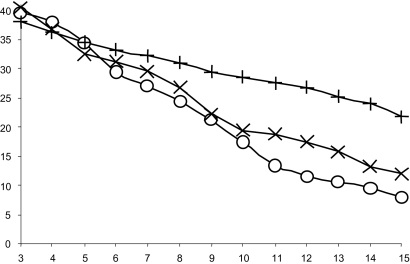
Mean topological recovery values obtained with respect to the number of present genes. The abscissa axis represents the number of concatenated genes that varies from 3 to 15. The curves indicate the variation of the Robinson and Foulds topological distance for the methods MRP (+), DNAML (×) and PEMV (○).

The results illustrated in Figure 3 show that PEMV has a better average accuracy when 6 to 15 genes were present. Obviously, the addition of a supplementary gene always improves the average accuracy of phylogenetic reconstruction because of a larger character sampling [25]. But in the same way, the growth of missing data affects the tree reconstruction. As shown, PEMV reduces the negative effect of missing data: The bigger the number of combined genes, the larger the difference between the phylogenies produced by the three methods. These results are in agreement with those found in [25]: The supermatrix methods used in this study usually provided a better topological recovery than the most popular supertree method MRP.

## Conclusion

The PEMV technique introduced in this article is a new efficient method that can be applied to infer large phylogenies from nucleotide sequences comprising missing data. The simulations conducted in this study demonstrated the usefulness of PEMV in estimating missing bases prior to phylogenetic reconstruction. Tested in the framework of the Kimura 2-parameter model [11], the PEMV method provided very promising results for the DNA sequences with 125 and 500 nucleotides as well as for long sequences comprising multiple genes [17]. The relative accuracy of the new method increases as the percentage of missing nucleotides increases. The deletion of missing sites, as it is done in the IMS method, or their estimation using PDMAB (two methods available in PAUP) can ignore or misinterpret important features of the data at hand. The application of PEMV to the multiple gene dataset showed that the new method can outperform the well-know supertree and supermatrix approaches. PEMV was included in the T-Rex package [15], which is freely available to researchers at the following URL: <http://www.labunix.uqam.ca/~makarenv/trex.html>.

In this paper, we presented PEMV in the framework of the Jukes-Cantor [10] and Kimura 2-parameter [11] models. It would be interesting to extend and test this probabilistic approach within more complex and more realistic models of sequences evolution, such as F84 [4], LogDet [24], or the Tajima and Nei model [27]. It is important to compare the results obtained using BioNJ to those produced using other distance-based methods of phylogenetic reconstruction, as for example, NJ [22], FITCH [6] and MW [14]. For the specific case of multiple gene phylogenies, it would be interesting to extend the model to take into account various substitution rates among sites.

## Figures and Tables

**Table 1:** Matrix **C** used to show the difference between the methods PEMV, IMS and PDMAB.

Matrix C	1	2	3	4	5	6	7	8
Sequence 1:	A	C	G	G	G	A	A	–
Sequence 2:	A	C	G	T	–	A	A	A
Sequence 3:	A	C	G	T	–	A	G	C

**Table 2:** Distribution matrix for the sequences 1 and 2 computed by PDMAB.

	A	C	G	T
A	4.0	0	0	0
C		1.0	0	0
G			1.5	1.5
T				0

**Table 3:** Results of the ANOVA tests carried out for the differences in the mean Robinson and Foulds topological recovery obtained using the methods PEMV, IMS and PDMAB.

Nb of taxa	125 bases				500 bases			
Missing %	8	16	24	32	8	16	24	32
10	0.099	3.33E-06	1.21E-10	3.91E-15	0.222	0.014	0.050	0.671
20	1.66E-05	4.14E-18	5.07E-37	3.48E-39	0.547	0.001	0.027	0.001
30	1.37E-12	4.52E-40	5.95E-66	2.94E-79	0.272	9.66E-06	1.65E-07	2.92E-08
40	7.91E-14	7.51E-69	5.50E-112	2.29E-140	0.343	1.90E-16	1.99E-20	3.81E-30
50	6.97E-12	2.19E-86	7.35E-162	9.18E-187	0.581	1.55E-20	5.74E-42	4.74E-54

## Appendix

### Theorem 1

For any sequence i, and any site k of the matrix C, such that c_ik_ is a missing nucleotide, the following equation holds: P_ik_(A) + P_ik_(C) + P_ik_(G) + P_ik_(T) = 1.

#### Proof

Replacing the sum *P**_ik_*(A) + *P**_ik_*(C) + *P**_ik_*(G) + *P**_ik_*(T) by the equivalent expression from Equation 1, we will need to prove that:

(6) $$\begin{array}{l} \frac{1}{N_{k}}(\sum_{\left\{j|C_{jk}=\text{A},\text{C},\text{G or T}\right\}}\delta_{ij}+\frac{1}{3}\sum_{\left\{j|C_{jk}\neq \text{A and}?\right\}}\left(1-\delta_{ij}\right)+\\ +\frac{1}{3}\sum_{\left\{j|C_{jk}\neq \text{C and}?\right\}}\left(1-\delta_{ij}\right)+\\ +\frac{1}{3}\sum_{\left\{j|C_{jk}\neq \text{G and}?\right\}}\left(1-\delta_{ij}\right)+\\ +\frac{1}{3}\sum_{\left\{j|C_{jk}\neq \text{T and}?\right\}}\left(1-\delta_{ij}\right))=1. \end{array}$$

Note that taking the sum over all the sequences such that *C**_jk_* ≠ A is equivalent to taking the sum over all the sequences such that *C**_jk_* = C, or *C**_jk_* = G, or *C**_jk_* = T. The similar considerations are true for the sum taken over all *C**_jk_* ≠ C, G, and T. Therefore, the left-hand part of Equation 6 can be rewritten as follows:

(7) $$\begin{array}{l} \frac{1}{N_{k}}(\sum_{\left\{j|C_{jk}=\text{A}\right\}}\delta_{ij}+\sum_{\left\{j|C_{jk}=\text{C}\right\}}\delta_{ij}+\\ \sum_{\left\{j|C_{jk}=\text{G}\right\}}\delta_{ij}+\sum_{\left\{j|C_{jk}=\text{T}\right\}}\delta_{ij}-\\ -\frac{1}{3}(3\sum_{\left\{j|C_{jk}=\text{A}\right\}}\delta_{ij}+3\sum_{\left\{j|C_{jk}=\text{C}\right\}}\delta_{ij}+\\ +3\sum_{\left\{j|C_{jk}=\text{G}\right\}}\delta_{ij}+3\sum_{\left\{j|C_{jk}=\text{T}\right\}}\delta_{ij}+)+\\ +\frac{1}{3}(3\sum_{\left\{j|C_{jk}=\text{A}\right\}}1+3\sum_{\left\{j|C_{jk}=\text{C}\right\}}1+\\ +3\sum_{\left\{j|C_{jk}=\text{G}\right\}}1+3\sum_{\left\{j|C_{jk}=\text{T}\right\}}1))=\\ =\frac{1}{N_{k}}(\sum_{\left\{j|C_{jk}=\text{A}\right\}}1+\sum_{\left\{j|C_{jk}=\text{C}\right\}}1+\\ \sum_{\left\{j|C_{jk}=\text{G}\right\}}1+\sum_{\left\{j|C_{jk}=\text{T}\right\}}1)=\frac{N_{k}}{N_{k}}=1 \end{array}$$

What had to be proved.
